# Dark-field radiography for the detection of bone microstructure changes in osteoporotic human lumbar spine specimens

**DOI:** 10.1186/s41747-024-00524-3

**Published:** 2024-11-04

**Authors:** Jon F. Rischewski, Florian T. Gassert, Theresa Urban, Johannes Hammel, Alexander Kufner, Christian Braun, Maximilian Lochschmidt, Marcus R. Makowski, Daniela Pfeiffer, Alexandra S. Gersing, Franz Pfeiffer

**Affiliations:** 1 Institute for Diagnostic and Interventional Neuroradiology, University Hospital, LMU Munich, Marchioninistr. 15, 81377 Munich, Germany; 2 Department of Diagnostic and Interventional Radiology, Klinikum Rechts der Isar, Technical University of Munich, Ismaninger Str. 22, 81675 Munich, Germany; 3 Chair of Biomedical Physics, Department of Physics, School of Natural Sciences, Technical University of Munich, James-Franck-Str. 1, 85748 Garching, Germany; 4 Munich Institute of Biomedical Engineering, Technical University of Munich, Boltzmannstraße 11, 85748 Garching, Germany; 5 Institute of Forensic Medicine, University Hospital of Munich, LMU Munich, Nußbaumstr. 26, 80336 Munich, Germany; 6 Munich Institute for Advanced Study, Technical University of Munich, Lichtenbergstr. 2a, 85748 Garching, Germany; 7 Department of Radiology and Biomedical Imaging, University of California, San Francisco, 505 Parnassus Avenue, M-391 San Francisco, CA USA

**Keywords:** Bone density, Cadaver, Lumbar vertebrae, Osteoporosis, Radiography

## Abstract

**Background:**

Dark-field radiography imaging exploits the wave character of x-rays to measure small-angle scattering on material interfaces, providing structural information with low radiation exposure. We explored the potential of dark-field imaging of bone microstructure to improve the assessment of bone strength in osteoporosis.

**Methods:**

We prospectively examined 14 osteoporotic/osteopenic and 21 non-osteoporotic/osteopenic human cadaveric vertebrae (L2–L4) with a clinical dark-field radiography system, micro-computed tomography (CT), and spectral CT. Dark-field images were obtained in both vertical and horizontal sample positions. Bone microstructural parameters (trabecular number, Tb.N; trabecular thickness, Tb.Th; bone volume fraction, BV/TV; degree of anisotropy, DA) were measured using standard *ex vivo* micro-CT, while hydroxyapatite density was measured using spectral CT. Correlations were assessed using Spearman rank correlation coefficients.

**Results:**

The measured dark-field signal was lower in osteoporotic/osteopenic vertebrae (vertical position, 0.23 ± 0.05 *versus* 0.29 ± 0.04, *p* < 0.001; horizontal position, 0.28 ± 0.06 *versus* 0.34 ± 0.04, *p* = 0.003). The dark-field signal from the vertical position correlated significantly with Tb.N (*ρ* = 0.46, *p* = 0.005), BV/TV (*ρ* = 0.45, *p* = 0.007), DA (*ρ* = -0.43, *p* = 0.010), and hydroxyapatite density (*ρ* = 0.53, *p* = 0.010). The calculated ratio of vertical/horizontal dark-field signal correlated significantly with Tb.N (*ρ* = 0.43, *p* = 0.011), BV/TV (*ρ* = 0.36, *p* = 0.032), DA (*ρ* = -0.51, *p* = 0.002), and hydroxyapatite density (*ρ* = 0.42, *p* = 0.049).

**Conclusion:**

Dark-field radiography is a feasible modality for drawing conclusions on bone microarchitecture in human cadaveric vertebral bone.

**Relevance statement:**

Gaining knowledge of the microarchitecture of bone contributes crucially to predicting bone strength in osteoporosis. This novel radiographic approach based on dark-field x-rays provides insights into bone microstructure at a lower radiation exposure than that of CT modalities.

**Key Points:**

- Dark-field radiography can give information on bone microstructure with low radiation exposure.
- The dark-field signal correlated positively with bone microstructure parameters.
- Dark-field signal correlated negatively with the degree of anisotropy.
- Dark-field radiography helps to determine the directionality of trabecular loss.

**Graphical Abstract:**

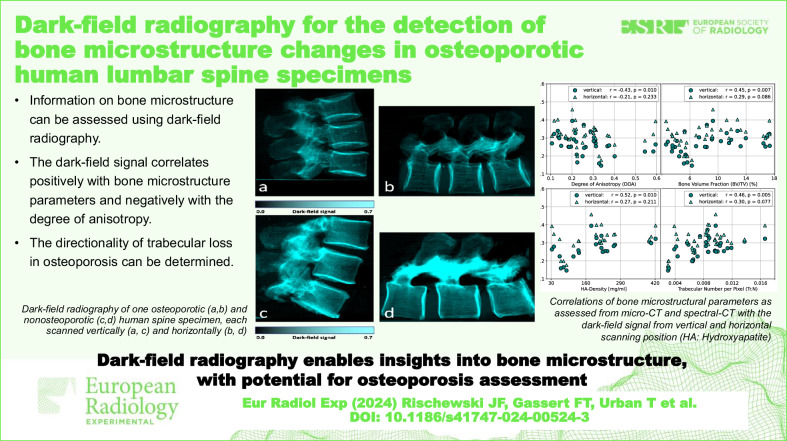

## Background

Osteoporosis is a systemic bone disease defined by low bone mass and a microstructure deterioration of skeletal tissue with a specific pattern, resulting in an increased risk of osseous fractures [1]. Osteoporotic fractures cause a socioeconomic burden as well as an increase in morbidity, mortality, and a decrease in quality of life [2, 3]. Therefore, early diagnosis is needed to timely initiate medical treatment to prevent poor outcomes in patients with high fracture risk [3]. However, osteoporosis is an underdiagnosed condition, and osteoporotic patients are commonly asymptomatic until a fracture occurs [4].

The standard screening tool for osteoporosis is dual-energy x-ray absorptiometry, which measures bone mineral density (BMD) [5]. Various studies have shown its insufficiency in osteoporosis assessment, emphasizing the need for additional parameters alongside BMD, such as bone microstructure and quality [6–8]. Including the dual-energy x-ray absorptiometry-derived trabecular bone score, which correlates with bone microstructure, improves identifying individuals at risk for future fracture [9]. Further microstructure quantification beyond BMD improves the assessment of bone strength significantly [7, 8, 10, 11]. Another screening tool is quantitative computed tomography (qCT). It yields a higher sensitivity for osteoporosis measuring volumetric BMD [12], but bears several disadvantages, such as higher radiation exposure, higher costs, and large precision errors [13].

Grating-based x-ray dark-field imaging [14, 15] is a novel modality recently introduced to osteoporosis research. The dark-field signal is generated by ultra-small-angle scattering at tissue interfaces, such as in trabecular bone [16]. This technique can obtain information on microstructures with the local scattering strength [17]. Until today, dark-field imaging has been mainly investigated for pulmonary imaging [15, 18–20], yielding good results in microstructure analysis for differentiating emphysematous from non-emphysematous lungs [15, 19]. Emphysematous tissue leads to less beam scattering due to fewer air-tissue interfaces in the lung parenchyma [15, 19]. Therefore, it is suggestive to use this imaging modality in osteoporosis diagnostics, due to the reduced trabecular number in osteoporotic bone [21]. As a non-tomographic modality, x-ray dark-field radiography can give structural information at low exposure to radiation [19, 22, 23]. The feasibility of distinguishing osteoporotic from non-osteoporotic spine samples with dark-field radiography has recently been shown [24]. Yet, this study concentrated solely on BMD measures to correlate with the dark-field signal and did not investigate the potential to draw conclusions on bone microstructure.

The aim of this study was to evaluate dark-field radiography for the assessment of the bone microstructure in human osteoporotic vertebrae. We investigated this by correlating the x-ray dark-field signal of *ex vivo* osteoporotic/osteopenic and non-osteoporotic/osteopenic lumbar vertebrae to bone quality parameters derived from micro-CT and spectral-CT scans.

## Methods

This prospective study was approved by the institutional ethics committee (Ethics Commission of the Medical Faculty, Technical University of Munich, Germany, Reference Number 70/17S, 26/08/2020) and was conducted according to the Declaration of Helsinki. Written informed consent was waived by the ethics committee.

### Cadaveric vertebral specimens

**Fig. 1:**
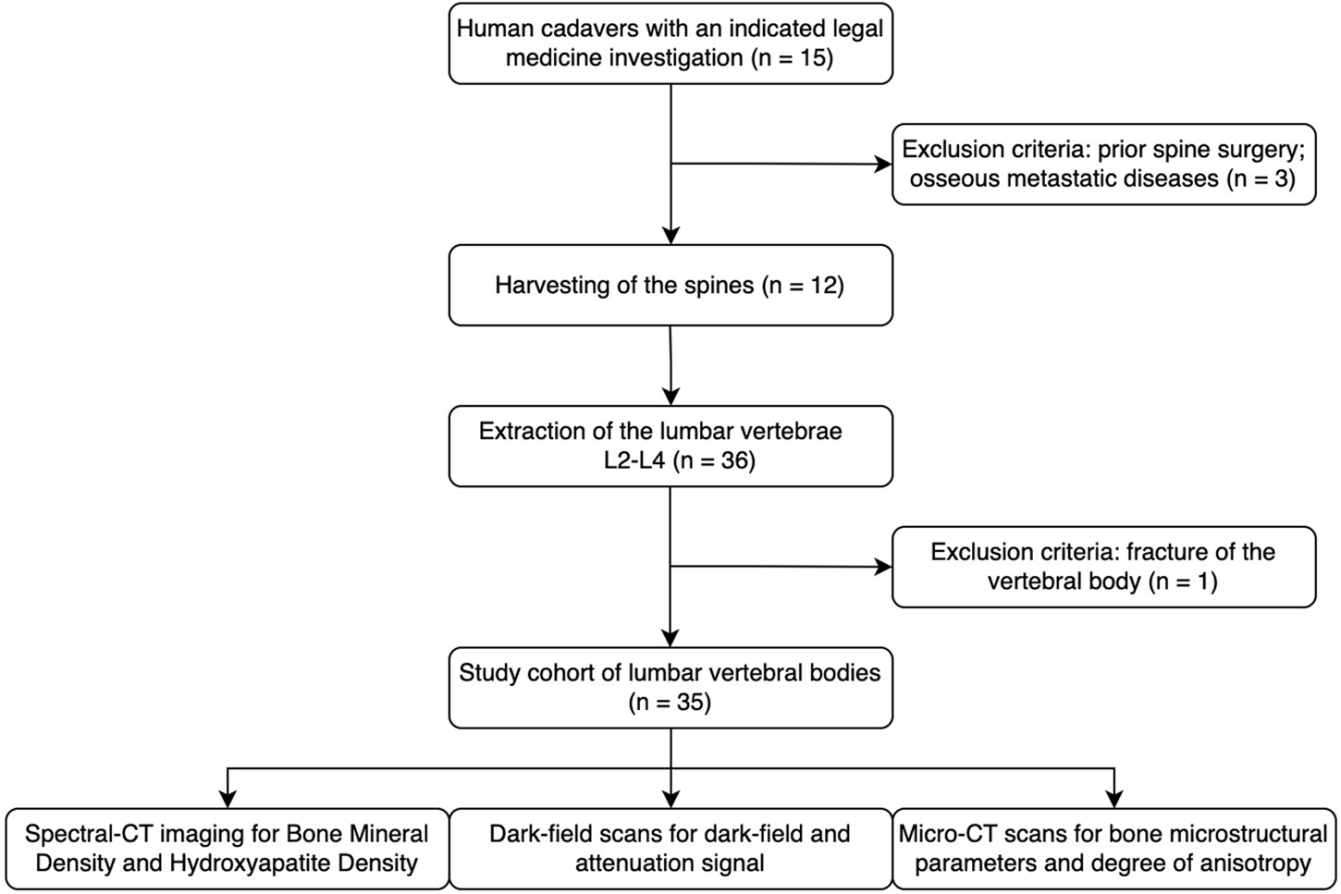
Flowchart of the Inclusion and exclusion criteria of the study cohort

Table 1 shows the demographics of the study cohort. A total of twelve human cadaveric lumbar spines (L2–L4) were harvested within 24 h after death in individuals with an indicated legal medicine post-mortem examination, yielding a total of 35 vertebrae (in one donor only L2 and L3 were harvested). Exclusion criteria for potential donors were osseous metastatic diseases, including hematological conditions, and prior spine surgery (*n* = 3). During harvesting of the spines, fractured vertebrae were excluded from this study (*n* = 1) (Fig. 1).

**Table 1:** Demographic characteristics of the study sample

Variables	Osteoporotic/osteopenic donors	Non-osteoporotic/osteopenic donors
Total number of donors	5	7
Females	4 (80%)	3 (43%)
Age (years) SD	72.2 ± 13.4	65.9 ± 15.1
Weight (kg)	71.6 ± 23.3	96.4 ± 19.5
Height (cm)	163.2 ± 7.6	166.6 ± 6.8
Body mass index (kg/m^2^)	26.6 ± 7.0	34.7 ± 6.1

Data are given as absolute numbers (%) or mean ± standard deviation

### Spectral-CT imaging

Dual-layer dual-energy CT (IQon Spectral CT, Philips Healthcare, Hamburg, Germany) of the vertebrae were performed with the following technical parameters: tube voltage 120 kVp; tube current 347 mA; collimation 0.6 mm; pixel spacing 0.3 mm; spiral pitch factor 0.39. Image data was reconstructed using a standard and bone filter with an axial slice thickness of 0.9 mm.

Asynchronously calibrated qCT was used to extract BMD values from regions of interest (ROI) manually segmented on sagittal reconstructions for the anterior part of the vertebra, calculated from HU, as described previously [25, 26]. Vertebrae with a BMD of < 120 mg/mL of bone mineral content were classified as osteoporotic or osteopenic (*n* = 14), while a BMD of ≥ 120 mg/mL was classified as non-osteoporotic/osteopenic vertebrae (*n* = 21) [27]. Additionally, hydroxyapatite density was measured by applying a material decomposition for bone and soft tissue from the spectral-CT images as previously described [28].

### Micro-CT scans

For the vertebral micro-CT scans, the tomographic setup consisted of a micro-focus x-ray tube, a photon-counting detector system (Varex Imaging, Hydra), and positioning devices mounted on an optical table [29]. The x-ray tube was operated on a tube voltage of 110 keVp and a current of 1,455 mA. The resulting isotropic voxel size was 88 µm, which is a sufficient resolution for analyzing the structure of human vertebral bone [8]. The sample was scanned in a plastic bag within a glass cylinder, placed 150 mm away from the x-ray source and 20 mm from the detector. In each scan, 1,600 images with an integration time of 0.2 s were taken over 360° of the sample. An in-house developed reconstruction software, named *pyCT*, based on Python, was used for reconstruction. A cone beam reconstruction geometry was assumed, and a Hamming filter was applied during the filtered back projection algorithm.

**Fig. 2:**
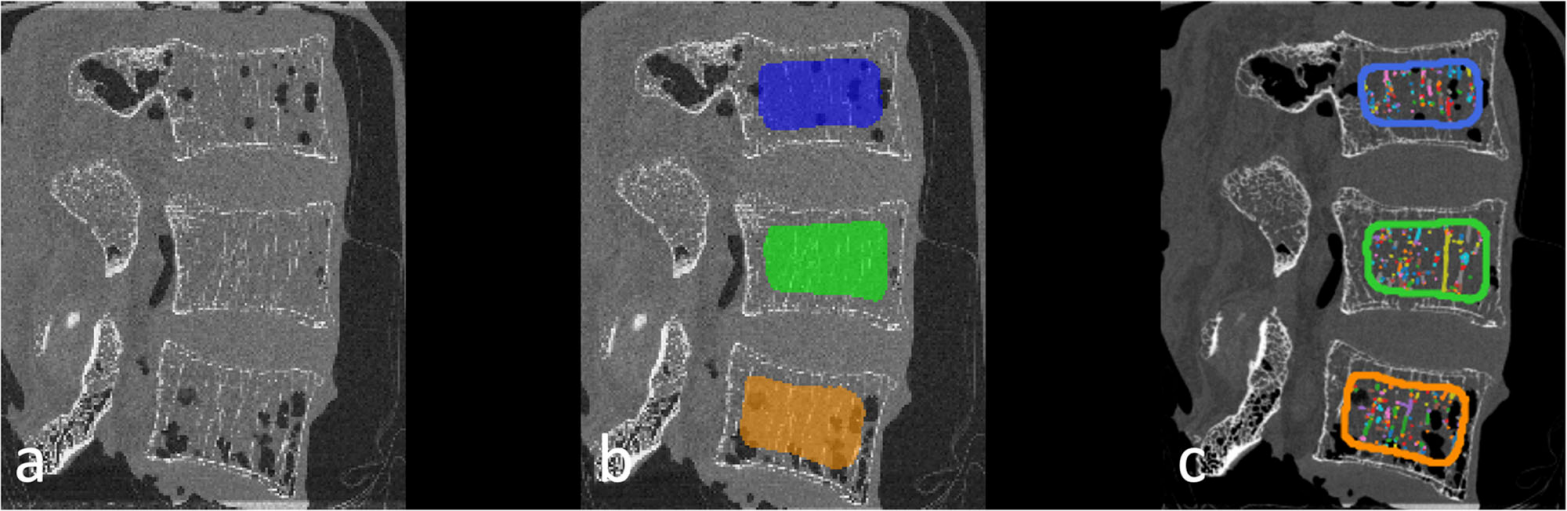
Spine segmentation and extraction of microstructural parameters. **a**–**c** Micro-CT-scan of an osteoporotic spine specimen of a 77-year-old female (bone mineral density = 65.75 mg/dL) with an illustration of the manually sampled segmentation masks (**b**). Derived from these masks, the trabeculae were automatically segmented (depicted as colorful dots and lines), and bone microstructural parameters were extracted using the Python library *Scikit-image* and *BoneJ* (**c**)

Manual ROIs of the entire trabecular region of the vertebral body were created by a radiologist with four years of experience in spine imaging. Bone trabeculae were automatically segmented from these ROIs, using Python, specifically with the *Scikit-image* library, for microstructure parameter extraction [30] (Fig. 2). For quantification of the trabecular microstructure, the bone volume fraction (bone volume divided by total volume, BV/TV) (%), bone surface fraction (bone surface divided by bone volume, BS/BV) (%), trabecular number (Tb.N), trabecular volume (Tb.V) (mm^3^) and trabecular thickness (Tb.Th) (mm) were obtained from the segmented trabeculae to quantify bone microstructure [31, 32]. Moreover, the degree of anisotropy (DA) was extracted using *BoneJ* (ImageJ, version 7.0.17) [33].

### X-ray dark-field imaging

**Fig. 3:**
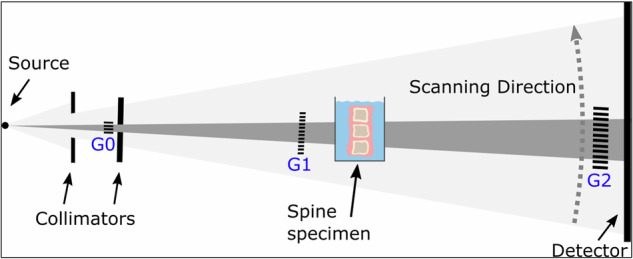
Illustration of the clinical dark-field radiography prototype system. The spine specimen is scanned in a water bath close to the grating G1 in a vertical position (as illustrated) and horizontal position (not shown). G0 and G2 schematically depict the absorption gratings used for generating the dark-field signal

The samples were imaged with a prototype for clinical dark-field chest radiography from the Technical University of Munich, as described in previous reports [15, 19, 24]. For the dark-field radiography system, a conventional medical x-ray tube (MRC 200 0508 ROT-GS 1003, Philips Medical Systems, Hamburg, Germany) and detector (PIXIUM 4343 F^4^, Trixell, Moirans, France) are combined with a Talbot-Lau interferometer with three gratings, enabling both the reconstruction of attenuation and dark-field images in one acquisition (Fig. 3).

A larger distance between the sample and analyzer grating than in previous pulmonary patient studies was needed to increase the sensitivity for osseous structures [24]. The potential effects of air around the sample were reduced by scanning the specimen in a water bath [24]. Reference scans of the water container without specimen were applied to reduce the influence of Compton scatter, and for the reduction of beam hardening influences, aluminum was used as an equivalent absorber material to apply a beam hardening correction [24, 34]. The reported values for the attenuation and dark-field signal represent the intensity (attenuation) and visibility (dark-field) of the signal relative to the measured intensity, respectively, visibility, in water on a logarithmic scale. As an example, the visibility/intensity of 0 in the sample translates into an equivalent signal strength as measured in water, the visibility of 1 represents a signal strength of 1/e compared to water.

**Fig. 4:**
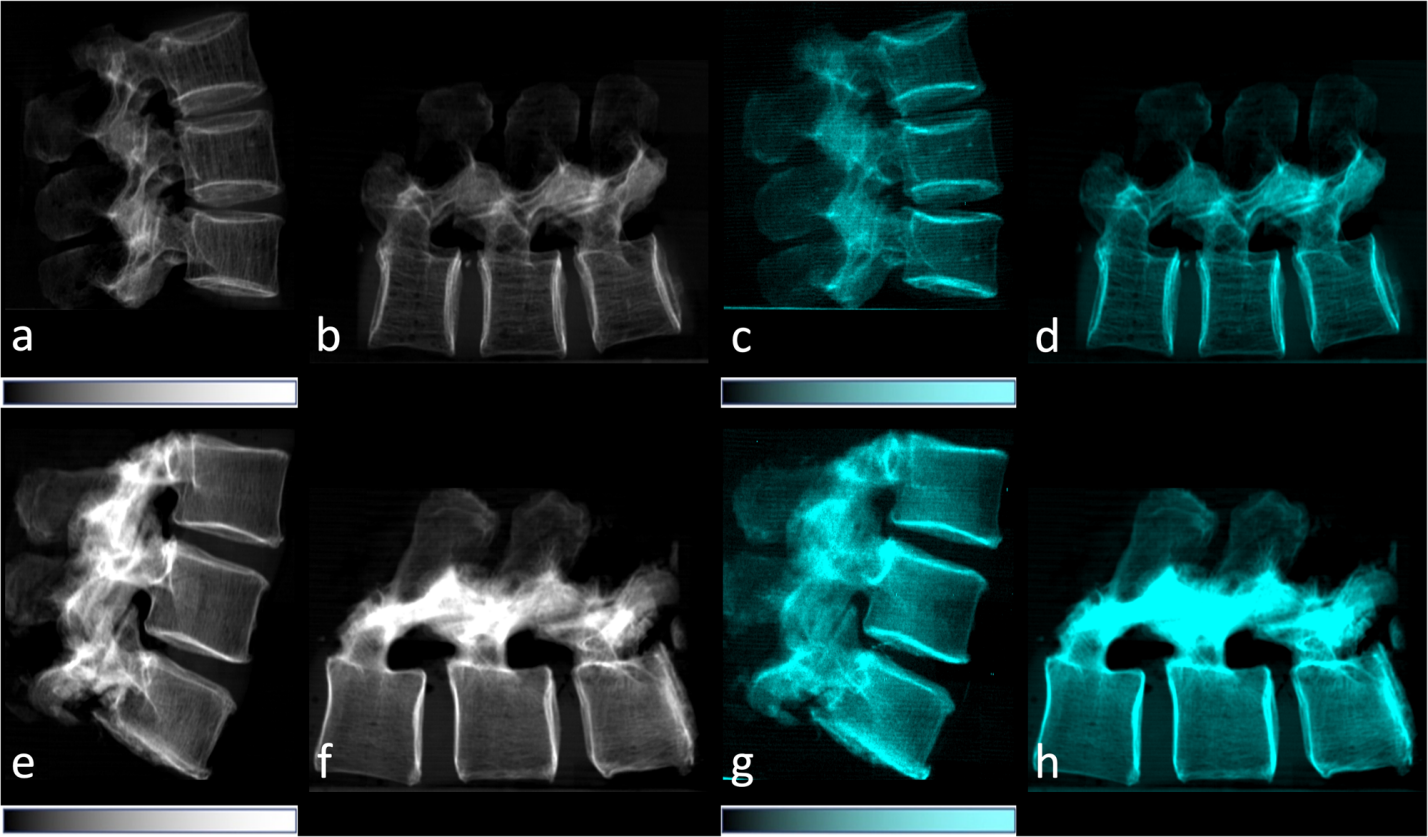
Lateral conventional attenuation (**a**, **b**, **e**, **f**) and co-registered dark-field (**c**, **d**, **g**, **h**) images of two spine specimens. Vertical (**a**, **c**, **e**, **g**) and horizontal (**b**, **d**, **f**, **h**) scans of the spine specimen of a 77-year-old female with osteoporosis (BMD = 65.75 mg/dL) (**a**–**d**) and a non-osteoporotic spine specimen (**e**–**h**) of a 61-year-old female (BMD = 169.38 mg/dL). BMD, Bone mineral density

The setup is only sensitive to structural elements parallel to the grating lamella. Since bone has trabecular structures both in lateral and cranio-caudal orientation, scans of the spines were performed in lateral orientation, in a vertical-standing, and a horizontal-lying position. The vertical-standing scan generated a dark-field signal from lateral trabeculae, and the horizontal-lying scan a dark-field signal from cranio-caudal trabeculae, respectively [16, 24, 35] (Fig. 4). Concerning the scattering caused by the cortical bone, it is not expected that it contributed significantly to the total amount of scattering, since it contains little tissue interfaces compared to the trabecular bone, which is responsible for creating the dark-field signal. The mean dose area product of the dark-field scans was 10.64 ± 0.03 dGy*cm^2^ (mean ± standard deviation) for the vertical scans and 10.62 ± 0.04 dGy*cm^2^ for the horizontal scans. Applying a dose conversion coefficient for lateral lumbar spine radiographs [36], the mean effective dose can be briefly estimated to 97.9 µSv for the vertical scans, and 97.7 µSv for the horizontal scans, respectively.

### Quantitative image evaluation

Python and SimpleElastix were used to co-register attenuation images with a rigid registration. On the horizontal and co-registered vertical attenuation images, ROIs were segmented manually in the anterior part of the vertebra using overlay images. Sclerotic or superimposed areas were excluded from segmentation. The same ROIs were applied to all attenuation and dark-field images of one spine specimen. The mean signal within each ROI was used to calculate quantitative values [24]. Due to technical limitations caused by the differing imaging modalities used for dark-field radiography and the qCT-based BMD measures, the same ROIs from this case could not be used for qCT but were covering a similar region of the vertebral bone as the ROIs taken to determine the BMD.

### Statistical analysis

Statistical analysis was performed using SPSS (version 29; IBM SPSS Statistics for macOS, IBM Corp., Armonk, NY, USA) using a two-sided significance level of 0.05 for all statistical tests. A Shapiro-Wilk test was performed to test for normal distribution of the data. To calculate differences between the osteoporotic/osteopenic and non-osteoporotic/osteopenic groups, a Student *t*-test was performed for the parameters attenuation and dark-field signal in vertical, horizontal, and the ratio of vertical/horizontal position, as well as for the parameters Tb.Th, Tb.V, Tb.N, and DA. For the parameters BMD, hydroxyapatite density, BV/TV, and BS/BV, we used a Wilcoxon Rank Sum Test. Correlations between microstructural parameters and x-ray dark-field imaging values were obtained with Spearman’s correlation. As primary analysis, based on prior studies on bone microstructure using micro-CT, we defined the comparison of the microstructural parameters BV/TV, Tb.N, Tb.Th, and DA between the two groups and their correlation with the dark-field signal [31, 32, 37]. As another parameter to estimate bone quality, we included the hydroxyapatite density in our primary analysis for correlation with the dark-field signal. For exploratory analysis, we investigated the group differences for BS/BV and Tb.V, the correlations of the dark-field signal with the BS/BV and Tb.V, and the correlations of the attenuation signal with all microstructural parameters. All values are given in mean ± standard deviation.

## Results

### Specimen

A total of 35 human cadaveric lumbar vertebrae (L2–L4, in one donor only L2 and L3) from 12 donors (7 females, 5 males) were analyzed; 14 were classified as osteoporotic/osteopenic and 21 as non-osteoporotic/osteopenic according to BMD measures performed on the samples. The donor’s average age was 68.5 ± 14.1 years; the average weight was 86.1 ± 23.8 kg, and the average body mass index was 31.3 ± 7.5 kg/m^2^.

### Quantitative analysis

Table 2 depicts the group differences between the osteoporotic/osteopenic and non-osteoporotic/osteopenic samples. Based on the BMD, the vertebrae were categorized into osteoporotic/osteopenic (*n* = 14) and non-osteoporotic/osteopenic vertebrae (*n* = 21) (osteoporotic/osteopenic group: 74.50 ± 25.80 mg/mL, non-osteoporotic/osteopenic group: 194.56 ± 23.45 mg/mL. The dark-field signal was significantly lower in osteoporotic/osteopenic vertebrae compared to non-osteoporotic/osteopenic samples for both the vertical (0.23 ± 0.05 *versus* 0.29 ± 0.04, *p* < 0.001) and horizontal orientation (0.28 ± 0.06 *versus* 0.34 ± 0.04, *p* = 0.003), as well as for the calculated ratio of vertical/horizontal dark-field signal (0.80 ± 0.07 *versus* 0.86 ± 0.07, *p* = 0.012). The attenuation signal was significantly lower for the osteoporotic/osteopenic specimens compared to the controls in vertical (0.21 ± 0.08 *versus* 0.33 ± 0.08, *p* < 0.001) and horizontal (0.21 ± 0.08 *versus* 0.32 ± 0.07, *p* < 0.001) orientation, while the calculated ratio of the signal showed no significant difference between the two groups (1.00 ± 0.07 *versus* 1.02 ± 0.08, *p* = 0.514). For the microstructural parameters, the Tr.N was significantly lower in osteoporotic/osteopenic vertebrae compared to the non-osteoporotic/osteopenic controls (0.005 ± 0.002 *versus* 0.010 ± 0.002, *p* < 0.001). Between the two cohorts, the osteoporotic/osteopenic specimen had a lower BV/TV (5.30 ± 2.53% *versus* 10.89 ± 4.39%, *p* < 0.001). No significant group difference was seen for Tb.Th (0.23 ± 0.08 *versus* 0.23 ± 0.06, *p* = 0.912). A higher DA was measured in the osteoporotic/osteopenic vertebrae group compared to the controls (0.34 ± 0.14 *versus* 0.22 ± 0.08; *p* = 0.002).

**Table 2:** Comparison of the quantitative parameters between the two study cohorts

Variables	Osteoporotic/osteopenic vertebrae (*n* = 14)	Non-osteoporotic/osteopenic vertebrae (*n* = 21)	*p*-value
Bone mineral density (mg/mL)	74.50 ± 25.80	194.56 ± 23.45	
Bone volume fraction (%)	5.30 ± 2.53	10.89 ± 4.39	< 0.001
Bone surface fraction (%)	5.86 ± 1.57	12.33 ± 3.92	< 0.001
Trabecular number	0.005 ± 0.002	0.010 ± 0.002	< 0.001
Trabecular thickness (mm)	0.23 ± 0.08	0.23 ± 0.06	0.912
Trabecular volume (mm^3)^	0.007 ± 0.004	0.007 ± 0.002	0.983
Degree of anisotropy	0.35 ± 0.14	0.22 ± 0.08	0.002
Hydroxyapatite density (mg/mL)	111.56 ± 73.33	266.72 ± 88.24	< 0.001
Dark-field signal vertical	0.23 ± 0.05	0.29 ± 0.04	< 0.001
Dark-field signal horizontal	0.28 ± 0.06	0.34 ± 0.04	0.003
Vertical/horizontal dark-field signal ratio	0.80 ± 0.07	0.86 ± 0.07	0.012
Attenuation signal vertical	0.21 ± 0.08	0.33 ± 0.08	< 0.001
Attenuation signal horizontal	0.21 ± 0.08	0.32 ± 0.07	< 0.001
Vertical/horizontal attenuation signal ratio	1.00 ± 0.07	1.02 ± 0.08	0.514

Data are given as mean ± standard deviation. The quantitative parameters were determined from the micro-CT scans, spectral-CT, and x-ray dark-field radiography. *CT* Computed tomography

The cohort showed a significantly lower value of hydroxyapatite density in the osteoporotic/osteopenic specimens (111.56 ± 73.33 mg/mL *versus* 266.72 ± 88.24 mg/mL, *p* < 0.001).

**Fig. 5:**
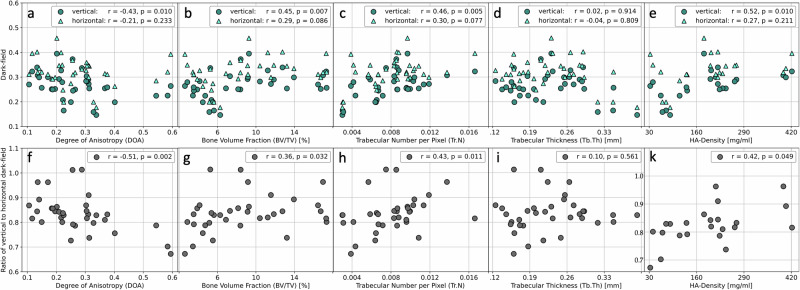
Primary analysis of correlations between the dark-field signal and bone quality parameters. Correlations of the microstructural parameters, degree of anisotropy (DA) and hydroxyapatite (HA) density with the dark-field signal from vertical and horizontal position (**a**–**e**) and with the calculated ratio from vertical/horizontal dark-field signal (**f**, **g**, **h**, **i**, **k**)

Figure 5 shows the primary data analysis for the correlation of the dark-field signal with bone microstructural parameters. We found a significant positive correlation of dark-field signal in a vertical position with the Tb.N (*ρ* = 0.46, *p* = 0.005), BV/TV (*ρ* = 0.45, *p* = 0.007), and hydroxyapatite density (*ρ* = 0.52, *p* = 0.010). The calculated ratio of vertical to horizontal dark-field signal correlated as well positively with the Tb.N (*ρ* = 0.43, *p* = 0.011), BV/TV (*ρ* = 0.36, *p* = 0.032) and hydroxyapatite density (*ρ* = 0.42, *p* = 0.049), whereas for the horizontal scanning position alone, no significant correlation was found for these parameters (Tb.N: *ρ* = 0.30, *p* = 0.077; BV/TV: *ρ* = 0.30, *p* = 0.086; hydroxyapatite density: *ρ* = 0.27, *p* = 0.211). No significant correlation was found between the Tb.Th and the dark-field signal measures (vertical: *ρ* = 0.02, *p* = 0.914; horizontal: *ρ* = -0.04, *p* = 0.809, signal ratio: *ρ* = 0.10, *p* = 0.561). A negative correlation between the DA and vertical dark-field signal (*ρ* = -0.43, *p* = 0.010), as well as with the vertical-to-horizontal signal ratio, was found (*ρ* = -0.51, *p* = 0.002), but not with horizontal dark-field signal (*ρ* = -0.21, *p* = 0.233).

The exploratory analysis of correlations between attenuation signal and microstructural parameters is displayed in Table 3.

**Table 3:** Correlations of the attenuation signal with BV/TV, Tb.N, Tb.Th, DA, and HA-density

	Attenuation vertical	*p*-value	Attenuation horizontal	*p*-value	Ratio vertical/horizontal signal	*p*-value
BV/TV (%)	*ρ* = 0.41	0.016	*ρ* = 0.44	0.009	*ρ* = -0.09	0.621
Tb.N	*ρ* = 0.41	0.016	*ρ* = 0.43	0.010	*ρ* = 0.02	0.910
Tb.Th (mm)	*ρ* = -0.02	0.917	*ρ* = 0.01	0.955	*ρ* = -0.16	0.374
DA	*ρ* = -0.42	0.012	*ρ* = -0.40	0.017	*ρ* = -0.20	0.241
HA-density	*ρ* = 0.62	0.002	*ρ* = 0.67	< 0.001	*ρ* = -0.34	0.116

Except for the Tb.Th, all measured parameters correlate significantly with the attenuation signal both in vertical and horizontal scanning orientation. There was no significant correlation between any parameter and the ratio of vertical/horizontal signal

*BV/TV* Bone volume fraction, *DA* Degree of anisotropy, *HA* Hydroxyapatite, *Tb.N* Trabecular number, *Tb.Th* Trabecular thickness

In the exploratory analysis of further microstructural parameters, no difference between the osteoporotic/osteopenic and non-osteoporotic/osteopenic samples for Tb.V (0.007 ± 0.004 mm^3^ *versus* 0.007 ± 0.002 mm^3^, *p* = 0.983) was found. The BS/BV showed a significant difference between the two groups (5.86 ± 1.57% *versus* 12.33 ± 3.92%, *p* < 0.001). Significant positive correlations between the BS/BV and the dark-field signal from vertical (*ρ* = 0.52, *p* = 0.001) and horizontal (*ρ* = 0.36, *p* = 0.035) scanning positions were found, as well for the calculated signal ratio (*ρ* = 0.41, *p* = 0.015). The attenuation signal also correlated positively with the BS/BV in both scanning positions (vertical, *ρ* = 0.46, *p* = 0.006; horizontal, *ρ* = 0.48, *p* = 0.003) but not for the calculated signal ratio. No significant association was found for Tb.V with dark-field or attenuation signals, respectively.

## Discussion

In this study, the potential of x-ray dark-field radiography to assess the bone microstructure in osteoporotic/osteopenic and non-osteoporotic/osteopenic human cadaveric spines was evaluated. The dark-field signal (acquired in vertical orientation), as well as the ratio of the vertically and horizontally acquired dark-field signal, correlated significantly with the degree of DA, Tr.N, BV/TV, and hydroxyapatite density.

Dark-field radiography uses the wave character of x-rays to measure small-angle scattering on tissue interfaces and generate a dark-field signal [14]. In previous pulmonary studies [15, 18–20], reduced air-tissue interfaces in emphysematous lung microstructure led to a reduced beam scattering and could, therefore, be detected using x-ray dark-field imaging [15, 19]. For bone, it was demonstrated that the signal is generated by interfaces between bone marrow and trabeculae [17, 24]. Bone strength is highly determined by the bone microstructure [11]. The most detailed determination can be made using a micro-CT system. Yet, this approach can only be applied in an *ex vivo* setting due to the setup and radiation doses [11]. Dark-field radiography, on the other hand, is a non-tomographic modality and, therefore, exposes the patient to significantly lower radiation doses compared to modalities such as CT. Prior studies on patients with our dark-field radiography system for imaging of the lung led to a radiation dosage exposure of 37 µSv for the posteroanterior images and 46 µSv for the lateral images [19, 23]. In our study, the effective dose can only be estimated briefly from the dose area product by applying conversion coefficients for lateral spine radiographs [36]. The estimated effective dose in our study was well below tomographic osteoporosis screening methods of the spine, with a mean value of 97.9 Sv for vertical and 97.7 µSv for horizontal scans, respectively. In comparison, a clinical single-slice qCT of the spine exposes the patients to an effective dose of 0.2–1.0 mSv, and a three-dimensional qCT to an effective dosage of 1.5 mSv [38]. Yet, standard qCT methods lack the ability to measure bone microstructure parameters [39]. To gain further knowledge on the exact effective radiation dosage applied in dark-field scans for the bone microstructure of the vertebrae, further studies as described by Frank et al [23] are needed.

Since the dark-field chest x-ray system is only sensitive to tissue interfaces parallel to the horizontal grating lamellae of the Talbot-Lau-Interferometer, but bone contains lateral and cranio-caudal trabeculae, the samples were scanned twice: vertically, creating a dark-field signal from lateral trabeculae, as well horizontally, which generates a signal from cranio-caudal trabeculae, respectively. Attenuation and dark-field signals can, therefore, be compared between both scan orientations and conclusions regarding the scattering strength of vertical and horizontal structures can be drawn. The lower dark-field signal measured in osteoporotic/osteopenic bone, especially in the vertical scan orientation, suggests a decrease of laterally oriented trabeculae that causes less scattering of the x-rays. These architectural changes are typical for osteoporotic bone, with a stronger loss of lateral trabeculae, while cranio-caudally aligned ones maintain more of their physiological structure [40–42].

A decrease in Tr.N and BV/TV led to a lower dark-field signal acquired from the vertical scanning position. This can be explained by fewer beam scattering due to the decreased number of lateral air-tissue interfaces in the osteoporotic/osteopenic samples. The rationale behind calculating the vertical-to-horizontal signal ratio and correlating it to the same microstructural parameters was conducted to emphasize how trabecular orientation affects the generated dark-field signal. Whilst the ratio from the attenuation images showed no difference in signal strength for the two scanning positions because the attenuation signal is determined by bone density but not by trabecular orientation, there was a positive correlation in the calculated dark-field signal ratio. The dark-field signal generated from lateral trabeculae decreased in osteoporotic/osteopenic samples, while the dark-field signal generated by cranio-caudal trabeculae did not decrease significantly.

Moreover, microstructural bone parameters that showed no significant difference between osteoporotic/osteopenic and non-osteoporotic/osteopenic samples were not significantly correlated with the dark-field signal. This supports our hypothesis that the microstructural changes in bone are responsible for dark-field signal alterations.

The higher DA measured in the osteoporotic/osteopenic samples can be explained by the typical architectural changes in osteoporotic bone, with an increased loss of laterally aligned trabeculae [40]. Conclusions on the DA of bone are especially valuable for determining bone stiffness and could, therefore, discover vertebrae at risk of fracture under moderate load [37, 43]. The negative correlation between dark-field signal and DA only in vertical but not in horizontal scan orientation suggests the feasibility of x-ray dark-field imaging to detect the typical changes of bone microarchitecture in osteoporotic vertebral bone. This is in line with the findings of prior studies, which could correctly predict trabecular orientation based on dark-field signals in bone samples [17, 22]. Differing from our approach, in the previous studies, small trabecular-only bone samples were scanned instead of whole vertebrae. Also, the x-ray dark-field system was not adapted to the human scale.

Interestingly, studies on x-ray dark-field radiography of the lung have demonstrated that the dark-field signal is affected less by superimposing soft tissue in direct comparison with the attenuation image [19]. This suggests a potential comparable behavior of dark-field signals in vertebral bone imaging *in vivo*. Yet, studies on a patient-scale are needed to verify this. If compared to prior *in vivo* studies on lung imaging performed with the same scanner that was used in this study, a longer exposition time or a stronger source could be needed for vertebral bone imaging due to the larger sample thickness when scanning a patient laterally. It can be expected that a more sensitive setup may be required, which can be achieved by choosing narrower grating lamellae. Dark-field radiography has the advantage, that it is an imaging modality that does not require the creation of an entirely new machine [44–46]. Both the x-ray tube and the detector used in our clinical prototype scanner are regular medical devices originally designed for x-ray imaging. Therefore, this technology has the potential to be implemented into regular x-ray units by modifying them with grating lamellae. As our scanner is a prototype, the exact setup to modify existing x-ray units has to be determined by future investigations.

This study has limitations. We did not correct for sample thickness influencing the measured signal. Due to the relatively small study size, studies on a larger scale are needed to verify our findings. We used an *ex vivo* approach on vertebral samples with little surrounding tissue. *In vivo* investigations are needed to evaluate the dark-field signal from the vertebral bone in patients. To enable a correlative analysis with micro-CT we used an *ex vivo* approach, which is a modality not feasible in patients due to the micro-CT setup and radiation doses applied. A further limitation would be the high average age of the study cohort, potential differing effects by a younger age on the dark-field signal could, therefore, not be accounted for. This is, on the one hand, due to the epidemiology of osteoporosis, affecting predominantly an elderly population, which ultimately led to a higher average donor’s age of osteoporotic/osteopenic spine specimens [2]. On the other hand, since this study relied on human tissue derived from donors at the Department of Legal Medicine, the amount of potential young spine specimen donors was limited. Due to technical restrictions from the studies’ set-up, DXA scans of the vertebral samples were not possible to perform. As a result, the trabecular bone score could not be used as a further parameter to be correlated with the dark-field signal. Additionally, it must be stated, that dark-field signal does not allow for a direct 3-dimensional measurement or finite-element-analysis, as it is a radiographic modality. However, an analysis of the vertebral three-dimensional structure seems to be feasible, since a significant correlation with the DA is shown in this study.

In summary, utilizing a prototype clinical x-ray dark-field radiography system, we observed a significant correlation between the dark-field signal and microstructure bone parameters of vertebral trabecular bone. This finding implies the potential application of dark-field imaging to draw conclusions on bone microstructure for predicting bone stability at a lower radiation exposure than in tomographic modalities. Dark-field imaging could, therefore, be a useful tool for assessing microstructural alterations of bone and become an important diagnostic component in osteoporosis imaging.

## Data Availability

Data generated or analyzed during this study are available from the corresponding author upon reasonable request.

## Abbreviations

BMD: Bone mineral density
BS/BV: Bone surface fraction
BV/TV: Bone volume fraction
CT: Computed tomography
DA: Degree of anisotropy
qCT: Quantitative computed tomography
ROI: Region of interest
Tb.N: Trabecular number
Tb.Th: Trabecular thickness
Tb.V: Trabecular volume
